# Convergent Evidence from Mouse and Human Studies Suggests the Involvement of Zinc Finger Protein 326 Gene in Antidepressant Treatment Response

**DOI:** 10.1371/journal.pone.0032984

**Published:** 2012-05-30

**Authors:** Ying-Jay Liou, Chien-Hsiun Chen, Chih-Ya Cheng, Shiow-Yi Chen, Tai-Jui Chen, Younger W-Y Yu, Fang-Shin Nian, Shih-Jen Tsai, Chen-Jee Hong

**Affiliations:** 1 Department of Psychiatry, Taipei Veterans General Hospital, Taipei, Taiwan; 2 Division of Psychiatry, School of Medicine, National Yang-Ming University, Taipei, Taiwan; 3 Institute of Brain Science, National Yang-Ming University, Taipei, Taiwan; 4 Institute of Clinical Medicine, School of Medicine, National Yang-Ming University, Taipei, Taiwan; 5 Institute of Biomedical Sciences, Academia Sinica, Taipei, Taiwan; 6 Graduate Institute of Chinese Medical Science, China Medical University, Taichung, Taiwan; 7 Institute of Bioscience and Biotechnology, National Taiwan Ocean University, Keelung, Taiwan; 8 Yu’s Psychiatric Clinic, Kaohsiung, Taiwan; Charité-Universitätsmedizin Berlin, Germany

## Abstract

**Objectives:**

The forced swim test (FST) is a commonly used model to predict antidepressant efficacy. Uncovering the genetic basis of the model may unravel the mechanism of antidepressant treatment.

**Methods:**

FVB/NJ (FVB) and C57BL/6J (B6) were first identified as the response and non-response strains to fluoxetine (a serotonin-specific reuptake inhibitor antidepressant) treatment in the mouse FST. Simple-interval (SIM) and composite-interval (CIM) mappings were applied to map the quantitative trait loci (QTLs) of the anti-immobility effect of fluoxetine in FST (FST_FLX_) in 865 male B6×FVB-F2 mice. The brain mRNA expressions of the gene with the maximum QTL-linkage signal for FST_FLX_ after the FST were compared between B6 and FVB mice and also compared between fluoxetine and saline treatment. The association of the variants in the human homologue of the mouse FST_FLX_-QTL gene with major depressive disorder (MDD) and antidepressant response were investigated in 1080 human subjects (MDD/control = 582/498).

**Results:**

One linkage signal for FST_FLX_-QTL was detected at an intronic SNP (rs6215396) of the mouse *Zfp326* gene (maximal CIM-LOD = 9.36). The *Zfp326* mRNA expression in the FVB thalamus was significantly down-regulated by fluoxetine in the FST, and the higher FVB-to-B6 *Zfp326* mRNA expressions in the frontal cortex, striatum and hypothalamus diminished after fluoxetine treatment. Two coding-synonymous SNPs (rs2816881 and rs10922744) in the human homologue of *Zfp326*, *ZNF326*, were significantly associated with the 8-week antidepressant treatment response in the MDD patients (Bonferroni-corrected p = 0.004–0.028).

**Conclusions:**

The findings suggest the involvement of the *Zfp326* and *ZNF326* genes in antidepressant treatment response.

## Introduction

Antidepressants are the main biological treatment for major depressive disorder (MDD). However, there are inter-individual differences in the response to antidepressant treatments: regardless of the kind of antidepressant used for initial treatment, 30–50% of patients do not respond sufficiently [1]–[5]. Although the mechanism and factors affecting individual responsiveness to antidepressants are yet unknown, several observations have demonstrated a high concordance of responsiveness to antidepressants in first-degree relatives [6]–[8], indicating that antidepressant efficacy is at least partly under genetic control [9].

The brain structure, neuronal signaling, circuits and genomic sequence between humans and mouse are highly conserved. With few exceptions, each human gene has a mouse ortholog and vice versa [10], [11]. Therefore, exploring the genetic mechanisms of mouse behavior may shed light on fundamental elements of human behavioral regulation [12], including responses to antidepressants.

It is thought that drug response is a complex and polygenic trait. To dissect the genetic bases of the trait, many studies have used quantitative trait locus (QTL) analysis to identify genomic loci associated with responsiveness to specific drugs [13]. The analysis is a statistical method that links phenotypic data and genotypic data. The QTL approach has successfully pinned down the *Usp46* gene that regulates the mouse baseline immobility time in the tail suspension test (TST) and forced swim test (FST) [14]. A similar approach also found the involvement of *Rgs2* gene in mouse anxiety [15]. The TST and FST are the most commonly used methods in animals to predict the efficacy of antidepressants. Crowley et al. identified two coding non-synonymous single nucleotide polymorphisms (Leu117Pro and Ser505Pro) in the mouse *vesicular monoamine transporter 2* gene that could be the QTL for the TST response to cilatopram treatment [16]. Liu et al. analyzed the NMRI×129S6 F2 mouse TST response to the tricyclic antidepressant imipramine and identified three suggestive chromosome regions (chromosome 1, 4 and 5) that might contain QTLs affecting the behavioral response [17]. Although several QTLs for the TST response to antidepressants have been identified, it is unclear whether the mouse FST response to antidepressants shares these QTLs. Most importantly, it remains unclear whether QTLs for the mouse response to antidepressants could be applied to predict human responses to antidepressants. In this study, we applied QTL analysis to localize the loci affecting the FST response to fluoxetine treatment in mice and used human samples to validate our findings.

## Materials and Methods

### Ethics

The study comprised an animal part and a human part. The animal part of the study was carried out in strict accordance with the recommendations in the Guide for the Care and Use of Laboratory Animals of the National Laboratory Animal Center, and was conducted with the approval of the Institutional Animal Care and Use Committee of Taipei Veterans General Hospital (approved on 2007/10/15) and National Taiwan Ocean University (approval ID: 96009). The animals were sacrificed by carbon dioxide narcosis, and all efforts were made to minimize suffering. The human part of the study was approved by the Institutional Review Board of Taipei Veterans General Hospital (VGHIRB No.: 95-11-07) and E-DA hospital (E-MRP-095-014), and written informed consent was obtained from the participants prior to enrollment.

### Animal and Drug Treatment

Animals were housed in plastic cages (five mice per cage) in a room maintained at a temperature of 24±1°C under a standard 12-hour light/dark cycle (lights on from 9∶00 a.m. to 9∶00 p.m.). Food and water were provided ad libitum. C57BL/6J (B6), BALB/cByJ (BALB) and FVB/NJ (FVB) were obtained from the National Laboratory Animal Center, Taiwan (http://www.nlac.org.tw/english/default.asp), and DBA/2N (DBA) and C3H/HeN (C3H) were purchased from BioLASCO Taiwan Co., Ltd (http://www.biolasco.com.tw/). F1 mice were generated by intercrossing the antidepressant-sensitive and antidepressant-insensitive strains, and F2 mice were obtained by intra-crossing the F1 mice. To avoid the potential influence of fluctuating estrogen and progesterone in the estrus cycle on the FST, only male mice were used throughout the study.

Fluoxetine hydrochloride (SIGMA) was prepared freshly and dissolved in deionized water in a volume of 4 ml/kg. Saline or fluoxetine was administered to the mice through intra-peritoneal injection (ip) 30 minutes prior to the FST. In studying strain differences in response to fluoxetine treatment, each animal of the same strain was randomly assigned to the following treatment groups: saline (4 ml/kg), 5 mg/kg, 10 mg/kg, or 20 mg/kg of fluoxetine. In the experiments for heritability and quantitative trait locus (QTL) analysis, all the F1 and F2 mice received 20 mg/kg of fluoxetine.

### Behavior Measurement: the FST

Each animal was tested in the FST at 8–10 weeks of age. The FST was conducted between 10∶00 and 16∶00 on two consecutive days. On day 1, animals were placed in an acrylic plastic box filled with water (23–24°C, 15 cm in depth) for 6 minutes. The total immobility time in the last 4 minutes (FST_BAS_) was recorded [18], [19]. Twenty hours later, saline or fluoxetine was administered to the mouse 30 minutes prior to the same procedure as that of day 1, and the total immobility time (FST_FLX_) was recorded. We used a digitalized apparatus, Method and System for Measuring Mobility of a Tested Animal (USA Invention Patent No.: US 7,121,229), to standardize the measurements of the FST. Agreement between the instrumental measurements and traditional naked-eye measurements for immobility time was high (Pearson correlation  = 0.932–0.957, p<0.001).

### Behavior Measurement: the Open Field Test

30 minutes after saline or fluoxetine (20 mg/kg) treatment mice were individually placed in an enclosed obstacle-free space (90×90×30 cm, L×W×H). The ambulatory distance (cm), time spent in traveling and traveling speed of the studied mice were measured by a video tracking system (Diagnostic & Research Instruments Co., Taiwan) for 6 minutes, corresponding to the observation period in the FST.

### Human Subjects and Assessments of Responses to Antidepressant Treatments

This study enrolled 582 patients with MDD and 498 controls. An author (YWY) who was blind to each subject’s genotype used the Schedules for Clinical Assessment in Neuropsychiatry (SCAN) [20] to assess the patients and made the diagnosis of MDD according to the DSM-IV. Patients who had additional Axis-I diagnoses (including schizophrenia, bipolar disorder, substance use disorder, anxiety disorder, etc.), pregnant patients, those who recently attempted suicide, and those with any major medical and/or neurological disorders were excluded. The control subjects screened by board-certificated psychiatrists were all free from major psychiatric illness. All the participants were aged between 20 and 65 years. Parts of the cases have been studied in our previous work [21]–[24].

In the MDD group, 262 patients were further evaluated in terms of their response to 8 weeks of treatment with fluoxetine or citalopram, both of which are selective serotonin reuptake inhibitors (SSRIs), to reduce pharmacological variability in terms of pharmacogenetic study of the antidepressants [25]. These patients had a minimum total score of 18 in the 21-item Hamilton Depression Rating Scale (HAMD) prior to treatment. The dose of both drugs started from 20 mg/day, which could be increased to 40 mg/day on the basis of clinical presentation and judgment. During the evaluation period, the use of any other psychotropic drugs was forbidden, except for night-time benzodiazepines for insomnia. For those patients with a past treatment history, a two-week drug-free interval was required before entering the study. Responders were defined as having a greater than 50% decrease in total HAMD score after 8 weeks of treatment. All the participants were Han Chinese.

### Marker Selection & Genotyping

For mapping of the mouse FST_FLX_, we used the ENU Mouse Gene Mapping Panel (http://nrpgm.sinica.edu.tw/en/serviceDetail.php?cNo=75), which consists of two single nucleotide polymorphisms (SNPs) on chromosome X and 197 SNPs on autosomal chromosomes to distinguish B6 from the FVB genetic background. In the analysis of human antidepressant response, two coding synonymous SNPs, rs2816881 C>A (Val412Val) and rs10922744 A>G (Glu505Glu), in the human *ZNF326* gene were selected from the CHB (Han Chinese in Beijing) population information listed in the International HapMap Project (http://hapmap.ncbi.nlm.nih.gov/index.html.en). The *ZNF326* gene is the human homologue of the mouse *Zfp326* gene. The two SNPs tag 100% of the genetic variations in *ZNF326* in the CHB population, under the marker selection criteria of no less than 10% of the minor allele frequency, greater than 0.8 in *r^2^* and two- and three-marker aggressive tagging with Haploview V4.1 (http://www.broadinstitute.org/haploview/haploview-downloads).

High-throughput MALDI-TOF mass spectrometry was adopted to genotype the SNPs. In genotyping the mouse samples, one B6, FVB and two F1 genomic DNAs were included as internal controls. For the human samples, 24 randomly selected subjects were re-genotyped for rs2816881 and rs10922744. The genotyping consistency rates for rs2816881 and rs10922744 were 100% and 96%, respectively.

### Quantification of mRNA Expression in the Mouse Brain

To quantify the *Zfp326* mRNA expression in different brain regions of the mouse, a new batch of B6 (n = 20) and FVB (n = 20) mice were subjected to the two-day FST and were also treated with saline or 20 mg/kg of fluoxetine 30 minutes before the 2^nd^ FST on the second day. At the end of the 2^nd^ FST, the animals were sacrificed immediately, and the frontal cortex, striatum, nucleus accumbens, thalamus, hypothalamus, amygdala and hippocampus were dissected and stored in RNA*later* (Ambion). Total RNA of individual brain regions was extracted with TRIzol (Invitrogen), treated with DNase I (Promega) and then transcribed to complimentary DNA (cDNA) using Moloney Murine Leukemia Virus (MMLV) reverse transcriptase according to the manufacturer’s instructions. RNA was quantified using absorption of light at 260 and 280 nm. The amounts of *Zfp326* and cyclophilin A cDNAs were measured using SYBR green-based real-time quantitative polymorphism chain reaction (RT-qPCR), which was performed using a Rotor-Gene 3000® (Corbett, Sydney, Australia), and the relative cDNA levels of *Zfp326* were normalized by the amount of *cyclophilin A* cDNA in the same sample. *Cyclophilin A* is an immediate factor of calcium/calmodulin signaling that is expressed ubiquitously [26]. The gene is expressed at a “high” level (>100 copies per cell) and its expression is less variable and more stable than that of GAPDH and beta-actin in the rat ischemia hippocampus and striatum [27], in experimental brain trauma in mice [28], and in a mouse model of kainite-induced mesiotemporal epilepsy [29]. It is also one of the most reliably expressed endogenous reference genes in the brain of those with Alzheimer’s disease [30]. Therefore, *cyclophilin A* appears to be a suitable internal or reference control in the quantitation of CNS mRNA expression [31], [32], was selected as an endogenous reference cDNA normalizer in this study.

A specific sample cDNA was used as a relative standard and inter-assay controller in each batch of RT-qPCR throughout the measurements of *Zfp326* and *cyclophilin A* cDNA. The primer sequences for *Zfp326* were zfp326-F: 5′-TGCAGATGATCACATGAT-3′ and zfp326-B: 5′-CCTGAGGGTGATCTTGAA-3′; for *cyclophilin A*, cyclophilin-A-F1∶5′-TATAAAGGAAGCCGCGGCGA-3′ and cyclophilin-A-B1∶5′-CTTTGTCTGCAAACAGCTCGA-3′. To obtain a reliable quantitative measurement of mouse brain cDNA, a linear standard curve was generated for each experiment plate. The standard curve was generated with serial 4-fold dilution to four dilution levels from one reference brain cDNA (*R^2^*>0.9). In all the experiments, the lowest and highest detection limits from the standard curves for *Zfp326* and *cyclophilin A* expression were as follows: *Zfp326*: lower  = 18.8∼19.3 Ct/upper  = 25.4∼25.8 Ct; *cyclophilin A*: lower  = 18.0∼18.6 Ct/upper  = 25.3∼25.6 Ct (Ct: the PCR cycle at which the sample reached the threshold). A sample with a Ct value residing in the linear range between the two detection limits was regarded as a reliable measurement. All samples were run in duplicate on the sample experiment plate. The intra-assay and inter-assay CVs for *Zfp326* and *cyclophilin A* mRNA were 0.8% and 2%, respectively. Data were derived from the mean of two independent amplifications.

### Statistical Analysis

The details of the analysis for the quantitative and qualitative traits measured in the study are summarized in the supplementary materials. In the QTL analysis for FST_FLX_, we first used simple interval mapping (SIM) in R/qtl (http://www.rqtl.org/) [33] to scan the potential region harboring the QTL for FST_FLX_. A “normal model” was used to scan the whole genome for normally-distributed phenotypes. However, for the phenotypes that deviated from a normal distribution, we performed the “non-parametric form of interval mapping” [33]. To control the residual genetic effect on SIM-detected signals, composite interval mapping (CIM) in R/qtl, a combination of SIM and multiple linear regression, was applied [34], [35]. The empirical genome-wide significant thresholds of 3.33 for SIM and 4.68 for CIM were obtained from 1000 permutation tests. The lowest LOD for suggestive linkage was set at 2.8 according to the guidelines proposed by Lander et al. [36].

In the C57×FVB F2 population, FST_FLX_ was positively correlated with FST_BAS_ (Pearson correlation coefficient  = 0.35, p<0.001). To reduce the confounding effect of FST_BAS_ on FST_FLX_, the standardized residual of FST_FLX_ (i.e., z_ FST_FLX_) was obtained with linear regression by treating FST_BAS_ as a covariate [17], [37]. The z_ FST_FLX_ was then subjected to QTL scanning with SIM and CIM. The empirical genome-wide significant thresholds of SIM and CIM for the phenotype were 3.38 and 4.08, respectively. The lowest LOD for suggestive linkage was also set at 2.8 [36].

In humans, the linkage between *ZNF326* rs2816881 and rs10922744 was estimated with Haploview V4.1. The difference in the haplotype frequency between groups was compared using SNPAlyze V3.5 (http://www.dynacom.co.jp/english/). The significance of the comparison was determined after 100000 permutation tests. As we tested two phenotypes (major depression diagnosis and treatment response after 8 weeks of antidepressant treatment) and two SNPs, the correction factor was 4, and a p-value lower than 0.0125 was regarded as significant.

## Results

### FST_BAS_ in Different Mouse Strains

There were significant differences in the FST_BAS_ among strains (*F*(4,227) = 27.84, p<0.001) (Table S1). Post hoc Scheffé’s procedure showed the following differences (p<0.05): BALB>B6, FVB, DBA, C3H; B6>C3H; FVB/NJ>C3H and DBA/2N>C3H. The FST_BAS_ of B6 and FVB were similar.

### Surveying FST_FLX_ in Different Mouse Strains

**Figure 1:**
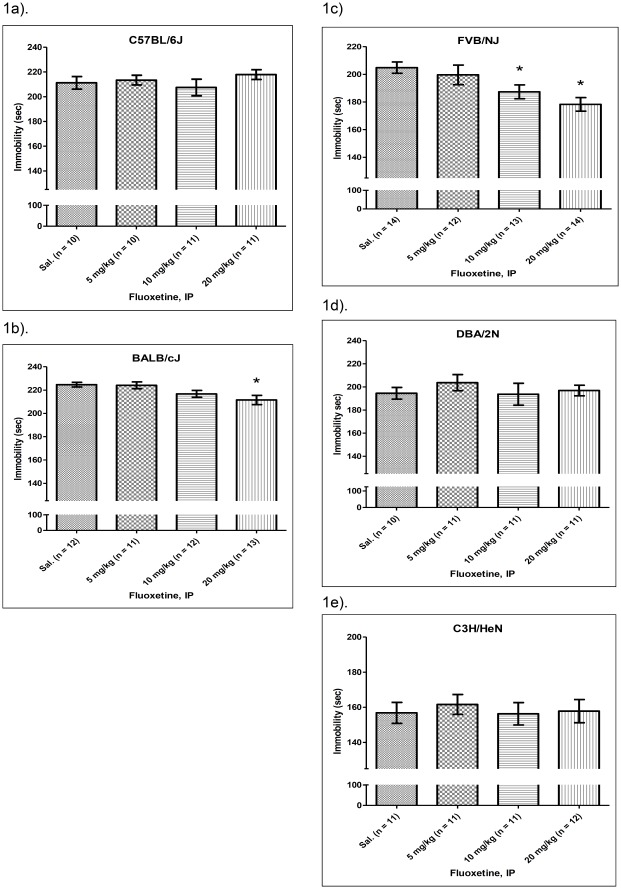
Strain-specific response to fluoxetine in mouse FST. Immobility time in the FST of B6 (1a), BALB (1b), FVB (1c), DBA (1d) and C3H (1e) mice after administration of different doses of fluoxetine. The FST was conducted 30 minutes after fluoxetine or saline injection (i.p.). “*” denotes a p-value lower than 0.05 compared with the saline group in the post hoc analysis. FST: forced swim test.

The mean FST_FLX_ in the B6 mice was not affected by administration of fluoxetine (*F*(3,38) = 0.76, p = 0.522, Figure 1a). However, a significant difference in the mean FST_FLX_ was observed in the BALB mice treated with various doses of fluoxetine (Figure 1b, *F*(3,44) = 4.198, p = 0.011). *Post hoc* Dunnett’s analysis indicated that the 20 mg/kg group had a lower FST_FLX_ than the saline group (p = 0.011). The FVB mice showed dose-dependent responses to fluoxetine in the FST (*F*(3,50) = 5.54, p = 0.002, Figure 1c). The 20 mg/kg group’s FST_FLX_ was significantly lower than that of the saline group (*post hoc* analysis p = 0.002), while the difference between the 10 mg/kg saline groups was borderline (p = 0.054). The FST_FLX_ of the DBA and C3H mice was not changed by the administration of different doses of fluoxetine (DBA: *F*(3,39) = 0.44, p = 0.723, Figure 1d; C3H: *F*(3,41) = 0.18, p = 0.931, Figure 1e). The survey suggested that the BALB and FVB mice were the fluoxetine-sensitive strains and B6 was the fluoxetine-insensitive strain in the FST.

### Confirmation of the Sensitive and Insensitive Strains in Response to Fluoxetine Treatment

In order to confirm the sensitive and insensitive strains in response to fluoxetine treatment, we used further batches of BALB, FVB and B6 mice and 20 mg/kg of fluoxetine to repeat the FST. In the FVB mice the fluoxetine group showed a shorter FST_FLX_ than the saline group (fluoxetine (n = 8) vs. saline (n = 8): 142.3±23.6 vs. 175.4±15.2 sec, two-tailed independent t test, p = 0.005). In the B6 mice, there was no significant difference in the FST_FLX_ between the fluoxetine and saline groups (fluoxetine (n = 8) vs. saline (n = 8): 210.2±14.1 vs. 212.4±6.0 sec, two-tailed independent t test, p = 0.688). The results of the BALB mice were inconsistent with those of the survey experiments (fluoxetine (n = 9) vs. saline (n = 8): 193.7±15.4 vs. 206.1±18.5 sec, two-tailed independent t test, p = 0.154). The results confirmed FVB to be the fluoxetine-sensitive strain and B6 to be the fluoxetine-insensitive strain in the FST.

### Open Field Test

In order to rule out the possibility that fluoxetine may induce hyperactivity [38], [39], open field tests were conducted 30 minutes after fluoxetine (20 mg/kg) administration in independent batches of experimentally naïve B6 and FVB mice. There was no significant difference in the mean traveling distance, time spent in traveling and the speed of traveling between the mice treated with saline and those treated with fluoxetine in both mouse strains (Table S2, all p>0.1).

### Heritability Study

**Figure 2:**
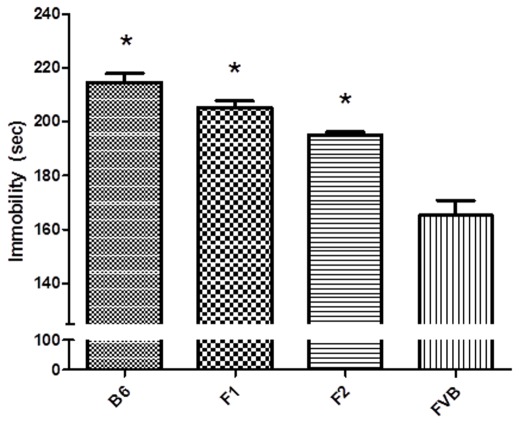
FST response to fluoxetine in B6, F1, F2 and FVB mice. Immobility time in the FST for B6 (n = 19), FVB (n = 22) and their F1 (n = 112) and F2 (n = 838) mice after fluoxetine treatment. The FST was conducted 30 minutes after fluoxetine (20 mg/kg) or saline injection (i.p.).“*” denotes a p-value lower than 0.05 in post hoc analysis compared with the FVB mice. FST: forced swim test; i.p.: intraperitoneal.

B6 (fluoxetine-insensitive strain) and FVB (fluoxetine-sensitive strain) mice were crossed to produce the F1 progeny. The F1 mice were intra-crossbred to generate the F2 progeny. The mean and variance of the FST_FLX_ of the B6, F1, F2 and FVB were as listed in Table S3. The mean FST_FLX_ were significantly different among the B6 (n = 19), F1 (n = 112), F2 (n = 865) and FVB (n = 22) mice (Figure 2, FST_FLX_: Kruskal-Wallis test, p<0.0001). The *post hoc* Mann-Whitney test revealed that the B6 mice had the longest FST_FLX_ (214.7±13.7 sec, least sensitive), F1 and F2 the intermediate FST_FLX_ (F1∶204.4±24.5 sec; F2∶195.0±32.2 sec), and FVB the shortest FST_FLX_ (165.1±26.6 sec, most sensitive) (B6> FVB, all p<0.0001, F1≥F2>FVB, all p<0.0001). As the F1 mice are genetically homogeneous, the broad-sense heritability (*H^2^*) for the F2 FST_FLX_ was 0.42 [(1039.7–602.4)/1039.7] [40]. The narrow-sense heritability (*h^2^*) due to all additive genetic contributions on FST_FLX_ variation of the F2 (V_A_/V_F2_) was 0.30. The heritability due to dominance (V_D_/V_F2_) was 0.06 (Table S3) [40].

### QTL Analysis for the FST Response to Fluoxetine

**Figure 3:**
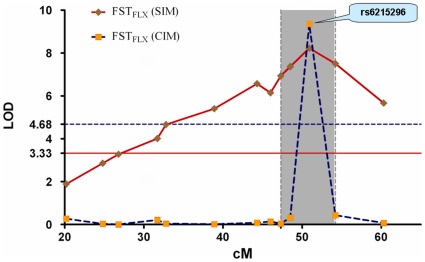
LOD scores for linkage for FST_FLX_. The solid red line at LOD  = 3.33 and the blue dashed line at LOD  = 4.68 denote the genome-wide significance threshold for FST_FLX_ (SIM) and FST_FLX_ (CIM), respectively. The gray zone indicates the 2-LOD confidence interval. FST: forced swim test; FST_FLX_: immobility in the mouse FST with fluoxetine treatment; LOD: logarithm of odds; SIM: simple interval mapping; CIM: composite interval mapping.

Using SIM, we identified 11 consecutive SNPs with LOD scores greater than the genome-wide significant threshold of 3.27 (Table 1). All the 11 SNPs are located in mouse chromosome 5, with a 2-LOD confidence interval (2-LOD CI) spanning the 47.3 cM to 54.2 cM region (Figure 3). Under the additive model, the 11 SNPs contributed 1.6%∼4.7% of the inter-individual variation in the FST_FLX_ (Table 1). The F2 mice with homozygous FVB alleles on these SNPs had a significantly shorter FST_FLX_ than the heterozygotes or homozygous B6 alleles (Table 1), suggesting that the F2 mice’s FST responsiveness to fluoxetine was affected by the alleles of the FVB mice. The results of the SIM and CIM for FST_FLX_ are compared and plotted in Figure 3. Although the LOD scores of some chromosome 5 markers obtained from the two algorithms were not always consistent, the maximum linkage signal detected by the SIM and CIM emerged on the same SNP rs6215296, with a LOD score of 8.21 in the SIM and 9.36 in the CIM. The inconsistency in the LOD scores obtained from the two programs for the other markers may arise from residual genetic influences on adjacent markers, an effect that is taken into account in the CIM but not in the SIM [35].

**Table 1:** Mouse single nucleotide polymorphisms (SNPs) that were associated with immobility time in the FST after fluoxetine treatment (FST_FLX_).

					FST_FLX_ (SD, N), seconds					
SNP ID	Location (cM)	LOD_(SIM)_	LOD_(CIM)_	Contribution (%)^a^	C57/C57	C57/FVB	FVB/FVB	P-value^b^	Response allele^c^	Nearestgene^d^
rs13459086	26.8	3.28	0.00	1.6	200.5 (30.0, 227)	195.1 (33.6, 415)	188.7 (32.8, 193)	0.001	FVB	*Gpr125*
rs13478271	31.7	4.01	0.21	1.8	200.2 (30.0, 220)	196.0 (33.0, 420)	187.4 (33.5, 196)	<0.001	FVB	*Pcdh7, LOC100041385*
rs6167151	32.8	4.67	0.04	2.2	201.4 (29.3, 218)	195.3 (33.4, 423)	187.2 (33.2, 193)	<0.001	FVB	*Tbc1d1, Ppia-ps22, LOC433894*
rs6366606	38.9	5.39	0.01	2.8	202.7 (28.6, 219)	195.0 (32.9, 423)	186.9 (34.5, 193)	<0.001	FVB	*Dcun1d4*
rs13478385	44.3	6.55	0.08	3.6	203.2 (28.2, 212)	195.8 (32.1, 413)	185.5 (35.6, 207)	<0.001	FVB	*Adamts3*
rs6232866	46.0	6.12	0.14	3.4	203.0 (28.2, 214)	195.8 (32.2, 415)	185.6 (35.7, 205)	<0.001	FVB	*G3pb2, Vdp*
rs13478403	47.3	6.91	0.04	3.0	203.2 (27.6, 205)	197.4 (29.8, 391)	188.0 (36.6, 191)	<0.001	FVB	*Fras1*
rs13478418	48.5	7.35	0.32	4.1	203.1 (28.4, 217)	196.1 (31.1, 410)	184.3 (36.8, 208)	<0.001	FVB	*LOC666417*
rs6215296	50.9	8.21	9.36	4.7	202.9 (28.2, 235)	197.4 (30.8, 389)	183.2 (36.6, 207)	<0.001	FVB	*Zfp326*
rs13478462	54.2	7.49	0.43	4.6	203.1 (27.5, 238)	196.2 (31.6, 391)	183.4 (36.9, 203)	<0.001	FVB	*Mn1, A230057G18Rik*
rs13478488	60.3	5.65	0.07	3.8	202.8 (27.3, 229)	195.5 (32.1, 409)	185.4 (36.8, 198)	<0.001	FVB	*EG665194, LOC100039529*

SD: standard deviation; cM: centi-morgan; LOD: logarithm (base 10) of odds; LOD_(SIM)_: the LOD scores derived from simple interval mapping; LOD_(CIM)_: LOD scores derived from composite interval mapping;

a The proportion of contribution the genetic polymorphism on the overall variation of the immobility time in FST after fluoxetine treatment.

b P-value for one way analysis of variance.

c The allele that shows significantly shorter immobility time in FST after fluoxetine treatment than the other allele.

d The list of the gene located in 1 mega-bases from the SNP.

In the F2 mice, about 13% of the individual variation in FST_FLX_ was correlated with their FST_BAS_ (FST_FLX_ vs. FST_BAS_: Pearson correlation coefficient  = 0.35, p<0.001). It is possible that the identified FST_FLX_-QTL is partly contributed to by the FST_BAS_. Therefore, the effect of FST_BAS_ was removed from each F2 mouse’s FST_FLX_ through linear regression. The standardized residuals of each F2 mouse’s FST_FLX_ (ie, z_ FST_FLX_) were then subjected to the SIM and CIM. One signal of z_ FST_FLX_ was linked to mouse chromosome 5 in the SIM and CIM (Figure S1). Again, both programs’ optimal results converged on rs6215296, with a LOD of 4.75 in the SIM and 4.80 in the CIM. The results were consistent with that using FST_FLX_ (Figure 3 for FST_FLX_), indicating that the SNP is a specific QTL for the mouse FST response to fluoxetine.

### Associations between Mouse Zfp326 Exonic Polymorphisms and Mouse FST Responses to Fluoxetine

**Figure 4:**
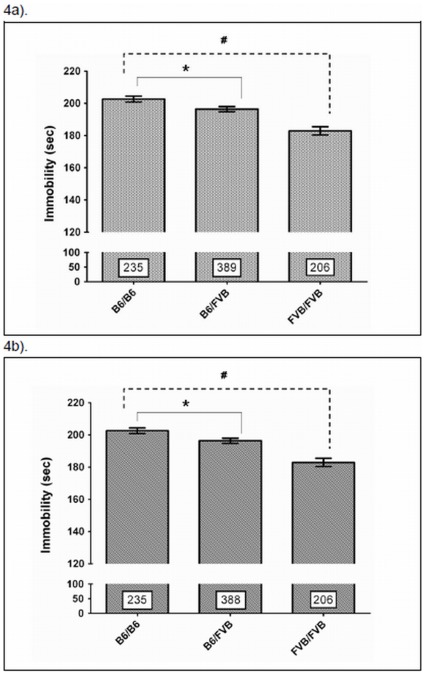
The association of mouse *Zfp326* function SNP with FST response to fluoxetine treatment. Genetic variations in *Zfp326*, rs33550587 (Asp494Gly) (4a) and rs13473815 (4b), are associated with the mouse response to fluoxetine in the FST. The B6×FVB-F2 mice were grouped according to genotype. * p<0.05; ^#^p<0.0001. The digits in the bars represent the number of animals in each group. *Zfp326*: zinc finger protein 326 gene; SNP: single nucleotide polymorphism; FST: forced swim test.

The maximum linkage signal for the FST response to fluoxetine is located on rs6215296 (Figure 3 and Figure S1), a SNP of the mouse *Zfp326* gene. There are two exonic SNPs in the mouse *Zfp326* that distinguish B6 from FVB mice: rs33550587 is a coding nonsynonymous SNP (Asp494Gly) in exon 12, while rs13473815 is a polymorphism in the 3-untranslated region (3′-UTR) (http://www.informatics.jax.org/strains_SNPs.shtml). *In silico* analysis predicted that different alleles of rs33550587 transcribe into different secondary structures of *Zfp326* mRNA (Figure S2a), and the same goes for rs13473815 (Figure S2b). To further study the effect of *Zfp326* variations on mouse responses to fluoxetine, the B6×FVB F2 mice were genotyped for the two *Zfp326* SNPs. For rs33550587, the F2 mice carrying one or two FVB alleles exhibited a shorter FST_FLX_ than the F2 carrying homozygous B6 alleles (*F*(2,827) = 21.9, p = 5.5×10^−10^, *post hoc* Dunnett t test: B6/B6 vs. B6/FVB, p = .032; B6/B6 vs. FVB/FVB, p = 3.8×10^−9^, Figure 4a). A similar finding was noted with rs1373815 (*F*(2,826) = 21.9, p = 5.6×10^−10^, *post hoc* Dunnett t test: B6/B6 vs. B6/FVB, p = 0.033; B6/B6 vs. FVB/FVB, p = 3.8×10^−9^, Figure 4b). The two SNPs rs33550587 and rs13473815 are in strong linkage disequilibrium (LD, absolute D’ = 1.0, *r^2^* = 1.0) with rs6215296, suggesting that the maximal detected LOD score of rs6215296 may reflect the effect of rs33550587 and rs13473815 on FST_FLX_ through inter-marker LD.

### Zfp326 mRNA Expressions in the Mouse Brain

**Figure 5:**
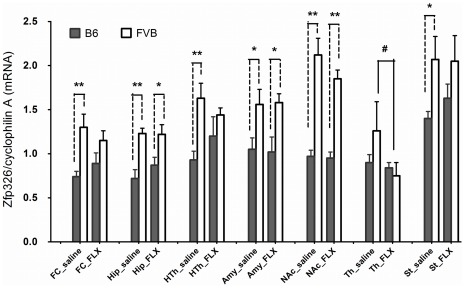
*Zfp326* mRNA expressions in different brain regions in B6 and FVB mice. The level of *Zfp326* mRNA was normalized by the level of cyclophilin A in the same region in each mouse. “**” and “*” represent a p-value lower than 0.01 and 0.05, respectively, comparing FVB with B6 in the same brain region with the Many-Whitney U test. “^#^” indicates a p-value lower than 0.05, comparing the same brain region of mice treated with saline or fluoxetine using the Many-Whitney U test. n = 8∼10 mice for each bar. FLX: fluoxetine. FC: frontal cortex; Hip: hippocampus; HTh: hypothalamus; Amy: amygdala; NAc: nucleus accumbens; Th: thalamus; St: striatum.

The mRNA levels of the reference gene *cyclophilin A* in the selected brain regions of B6 and FVB were not regulated by the treatment with fluoxetine in the FST (Table S4), indicating that *cyclophilin A* is an appropriate endogenous reference gene for use in this study. Figure 5 shows the *Zfp326* mRNA expression in different brain regions of B6 or FVB mice after 20 mg/kg of fluoxetine treatment. Significant inter-strain differences in *Zfp326* mRNA expression between B6 and FVB were noted in the frontal cortex, hippocampus, hypothalamus, amygdala, nucleus accumbens and striatum (Mann-Whitney U test, all p<0.05). However, after fluoxetine treatment, the inter-strain difference in the frontal cortex, hypothalamus and striatum disappeared (Figure 5). Furthermore, in FVB, the *Zfp326* mRNA levels in the thalamus were significantly lower in the fluoxetine group than in the saline group (Mann-Whitney test, p = 0.027).

### Association between Human ZNF326 Variations and Responses to SSRI Antidepressant Treatments

There are two coding-synonymous SNPs in the human *ZNF326* gene (rs2816881 (Val412Val) and rs10922744 (Glu505Glu)). They tag 100% of the overall *ZNF326* genetic variations in the CHB population recruited in the international HapMap project (http://hapmap.ncbi.nlm.nih.gov/). In the study, the gender proportions and mean age of the MDD and control groups were similar (gender (male/female): MDD vs. control  = 238/344 vs. 220/278, p = 0.276; age (SD): MDD vs. control  = 44.3 (16.4) vs. 43.1 (12.6), p = 0.218). There was no significant difference between the responders and non-responders in terms of gender distribution, antidepressants (fluoxetine or citalopram), mean age, duration of current episode, number of previous episodes and mean baseline HAMD scores (Table S5, all p>0.05).

Table 2 shows the genotype and allele distributions in the study subjects. The genotype distributions of the two SNPs in the MDD and control subjects were in Hardy-Weinberg equilibrium. The genotype and allele distributions of the two *ZNF326* SNPs were similar between the MDD patients and controls, but were significantly different between the responders and non-responders. The A-carrier rate of rs2816881 was higher in the responders (15.3%) than in the non-responders (3.3%) (Fisher’s exact p = 0.003, odds ratio (OR) for being the responder  = 5.20, 95% CI = 1.53–17.69). Similarly, the G-carrier rate of rs10922744 was higher in the responders (15.7%) than in the non-responders (3.4%) (Fisher’s exact p = 0.002, OR for being the responder  = 5.34, 95% CI = 1.57–18.12). Haplotype-based analysis for rs2816881–rs10922744 did not further increase the significance (data not shown) because they are highly linked (absolute D’ = 1.0, *r^2^* = 1.0). The association between the two *ZNF326* SNPs and antidepressant treatment response survived the corrections for multiple comparisons.

**Table 2:** Genotype and allele distribution of *ZNF326* polymorphisms in the controls, and in the patients with major depressive disorder and their responses to 8-weeks’ antidepressant treatment.

		Phenotype			
		Diagnosis		8-week treatment response	
SNP	Genotype/allele	MDD (%)	Control (%)	Responder (%)	Non-responder (%)
rs2816881	CC	488 (84.3)	437 (81.7)	145 (84.8)	87 (96.7)
(Val412Val)	CA	84 (14.5)	94 (17.6)	23 (13.5)	3 (3.3)
	AA	7 (1.2)	4 (0.7)	3 (1.8)	0 (0.0)
	P-value_(genotype)_	0.305		**0.007** ^#^	
	C allele	1060 (91.5)	968 (90.5)	313 (91.5)	177 (98.3)
	A allele	98 (8.5)	102 (9.5)	29 (8.5)	3 (1.7)
	P-value_(allele)_	0.377		**0.0017** ^#^	
rs10922744	AA	480 (83.9)	432 (81.5)	145 (84.3)	86 (96.6)
(Glu505Glu)	AG	85 (14.9)	95 (17.9)	24 (14.0)	3 (3.4)
	GG	7 (1.2)	3 (0.6)	3 (1.7)	0 (0.0)
	P-value_(genotype)_	0.232^#^		**0.005** ^#^	
	A allele	1045 (91.3)	959 (90.5)	314 (91.3)	175 (98.3)
	G allele	99 (8.7)	101 (8.5)	30 (8.7)	3 (1.7)
	P-value_(allele)_	0.475		**0.001** ^#^	

* The P-value in boldface indicates the significance survived after correction for multiple comparisons (adjusted significant threshold with Bonferroni’s procedure: P<0.0125). “#” denotes the p-value obtained from Fisher exact test.

## Discussion

### Identification of the QTL for Mouse Sensitivity to Fluoxetine

FST and TST are usually served as convenient animal models for predicting the efficacy of antidepressants. Several mouse chromosome regions (chromosomes 1, 4, 5, 7, 12 and 19) have been linked to the mouse response to antidepressants in the TST using QTL mapping [16], [17]. However, the genetic determinants underlying the response to antidepressant in the mouse FST have not been extensively studied. In this study, we applied QTL mapping to narrow down the genetic factors affecting the mouse FST response to fluoxetine in 865 male B6×FVB F2 mice. Both the SIM and the CIM algorithms consistently revealed linkage signals in the 26.8–60.3 cM region of chromosome 5 (2-LOD CI  = 47.3–54.2 cM) (Table 1 & Figure 3). The results suggest that the region contains the QTL of the mouse FST response to fluoxetine. Interestingly, Liu et al. reported that D5Mit41 (0.25 cM away from rs6215296) was a suggestive QTL (LOD  = 2.9) for mouse sensitivity to imipramine treatment in the TST [17]. The close proximity of rs6215296 and D5Mit41 indicates that the 47.3–54.2 cM region of chromosome 5 contains shared QTL(s) modifying responses to antidepressants in the TST and FST.

There are five other suggestive QTLs thought to be associated with the anti-immobility effect of antidepressant in the TST. Crowley et al. reported that one significant QTL on mouse chromosome 19 (D19Mit71) and two suggestive QTLs on chromosome 7 (D07Mit259) and chromosome 12 (D12Mit118) were associated with mouse responses to citalopram, another SSRI antidepressant, in the TST [16]. The other suggestive QTLs (D1Mit410 and D4Mit204) for response to imipramine in the TST were reported in the work of Liu et al. [17]. However, we did not obtain significant LOD scores in those regions in this study. The inconsistencies among studies might arise owing to the different parental strains (ex, Balb/cJ×A/J vs. NMRI×129S6 vs. B6×FVB), behavioral paradigms (TST vs. FST), sample sizes and/or the use of antidepressants (e.g., imipramine, citalopram or fluoxetine).

### Strain Difference and Fluoxetine Treatment on Zfp326 Expression in the Brain

The maximum linkage signal for the FST response to fluoxetine is located on rs6215296. Since the SNP is located on mouse *Zfp326* gene, we looked into *Zfp326.* Except for limited evidence of involvement in neuronal differentiation [41], the function of *Zfp326* has rarely been explored. We investigated the expression profiles of *Zfp326* in the brain of FVB and B6 mice. The *Zfp326* mRNA levels of the FVB mice were significantly higher than those of the B6 mice in the frontal cortex, hypothalamus and striatum, but the inter-strain difference became insignificant after fluoxetine treatment (Figure 5). Furthermore, in the thalamus of FVB, the *Zfp326* mRNA expression was down-regulated by fluoxetine treatment (Figure 5). Interestingly, the four regions are thought to be involved in the cognitive aspect (feelings of worthlessness and guilt), neuro-vegetative signs and hedonic deficit of depression [42]. The results that *Zfp326* mRNA levels are different in some brain areas of B6 and FVB mice and that *Zfp326* mRNA can be regulated by fluoxetine in some brain areas may suggest a role of *Zfp326* in regulating mouse sensitivity to fluoxetine and partly explain the difference in sensitivity to fluoxetine treatment between B6 and FVB mice. In addition, two genetic variants in *Zfp326*, one with amino acid change (rs33550587, Asp494Gly) and one in the 3′-UTR region (rs13473815), are associated with the mouse FST response to fluoxetine (Figure 4). These findings provide further evidence supporting the involvement of *Zfp326* in the mouse FST response to fluoxetine and explaining the discrepancy in sensitivity to fluoxetine between B6 and FVB mice.

### Association between ZNF326 Variations and Human Responses to Antidepressants

Since the FST is a widely used animal model in predicting antidepressant efficacy, it is imperative to examine the role of human homologue of mouse *Zfp326*, *ZNF326*, in predicting the efficacy of fluoxetine in humans. We found that rs2816881-A and rs10922744-G and the A–G haplotype were significantly associated with a favorable response in patients with MDD after 8 weeks of antidepressant treatment (Table 2). The effect of the two SNPs on *ZNF326* function is unclear and remains to be studied. Both rs2816881 and rs10922744 are coding synonymous SNPs and do not change the corresponding amino acid in the ZNF326 protein sequence. According to the finding of Nembaware et al.’s study, rs2816881 and its surrounding nucleotide sequence constitute a splicing regulatory sequence located at the exonic splicing enhancer elements of *ZNF326* [43]. Meanwhile, *in silico* prediction shows that both SNPs may have an effect on the *ZNF326* RNA secondary structure (Figure S2). In addition, rs2816881 and rs10922744 are in strong linkage disequilibrium with another *ZNF326* SNP, rs11808927, a *cis*-acting SNP involved in *ZNF326* mRNA expression in human liver (http://hapmap.ncbi.nlm.nih.gov/index.html.en) [44]. Therefore, rs2816881 and rs10922744 may affect *ZNF326* mRNA expressions through changing *ZNF326* RNA secondary structure, or disrupting normal gene splicing and causing aberrant splicing of either a proportion or all of the transcripts produced [43]. Interestingly, a recent genome-wide association study conducted in the GENDEP project reported that three SNPs (rs2136093, rs6701608 and rs2136094), about 0.20 Mb away from *ZNF326* in human chromosome 1 were in suggestive association (p = 3.82–5.56×10^−7^) with antidepressant responses [45]. Based on the convergent evidence obtained from mouse FST_FLX_-QTL mapping, human study in Uher et al. [45], and our results, it is highly possible that zinc finger protein 326 is involved in regulating the effect of fluoxetine in mice and humans.

### The Function of Zfp326 Needs Further Exploration

The *Zfp326* gene encodes a protein that contains two C2H2-type zinc finger motifs and glutamic acid-rich domains in the C-terminal region [46]. It exhibits DNA-binding activity in a zinc-dependent manner and plays a role in regulating cell growth [46]. *Zfp326* is thought to play a role in neuronal differentiation because its expression in the neuro-epithelium of the brain and neural tube in E11.5 embryos increased and its mRNA and protein were transiently elevated in cells treated with retinoic acid [41]. Recently, another human zinc finger protein *ZNF804A* gene, which also encodes a protein product with a C2H2-type domain like *Zfp326*, was reported to be associated with schizophrenia and a broader psychosis phenotype [47], [48]. However, detailed information about the *Zfp326* gene and its mechanism of functioning is still unknown, and the role of the gene in the pathogenesis of depression and antidepressant effect requires further characterization.

### Discrepancies Among Inbred Mouse Strains in Response to Antidepressants

In the first part of the study, we identified B6 as the fluoxetine-insensitive strain and FVB as the fluoxetine-sensitive strain. In a study of such a huge scale, the use of an automatic recording device is an important factor in achieving significant linkage results. Crowley et al. suggested that the use of an automatic recording device for scoring rodent behavior in the FST had many advantages over manual scoring [49], which included consistency and reliability across different experiments, raters and laboratories [49]. However, the applicability of an automatic device for the FST requires validation through contrasting with manual scoring procedures [49]. Prior to the commencement of the study, we proved that the correlation between manual and device scoring for the FST was high (Pearson correlation coefficient  = 0.93–0.96, p<0.001). In order to confirm the sensitive and insensitive strains for the subsequent heritability test and QTL analysis, replication experiments using a fixed dose of 20 mg/kg of fluoxetine and independent batches of B6, BALB and FVB mice were conducted. The results confirmed that the FVB and B6 were respectively sensitive and insensitive to 20 mg/kg of fluoxetine treatment in the FST (result 3.3). Possible psychostimulant or hyperactivity-inducing effects of fluoxetine [38] on the FST were ruled out, because neither the B6 nor FVB mice displayed increased activity in the open field test 30 minutes after fluoxetine treatment (Table S2).

### Conclusion

Through genotyping of 199 SNPs of 865 male B6×FVB F2 mice and applying SIM and CIM analysis, we localized the trait to the 47.3–54.2 cM region of mouse chromosome 5. Inside the region, a rarely studied gene, *Zfp326*, deserves special attention, because (1) its expression can be regulated by fluoxetine in the thalamus of FVB, (2) its expression differs between B6 and FVB mice in some brain areas and the discrepancies can be altered by fluoxetine, and (3) the SNPs in *Zfp326* were found to be associated with sensitivity to fluoxetine in the F2 mice. Most importantly, polymorphisms of the human homologue of *Zfp326*, *ZNF326,* were found to be associated with therapeutic response to SSRI treatment in patients with major depressive disorder. By studying the function of *Zfp326* or *ZNF326*, we may unravel the mechanism of antidepressant action and gain further insight into the pathology of major depressive disorder.

## Supporting Information

Figure S1 **LOD scores for linkage for z_ FST_FLX_.** The solid blue line at LOD  = 3.38 and green dash line at LOD  = 4.08 denotes the genome-wide significant threshold for z_ FST_FLX_ (SIM) and z_ FST_FLX_ (CIM) respectively. The gray zone indicates the 2-LOD confidence interval. z_ FST_FLX_: the baseline adjusted FST immobility with fluoxetine treatment; LOD: logarithm of odds; SIM: simple interval mapping; CIM: composite interval mapping.
(DOC)

Figure S2 **Cartoons indicate that different alleles ofrs33550587 (G>A) (5a) and rs13473815 (G>A) may have different effects on the secondary structure of transcribed** ***Zfp326*** **mRNA (5b).**
(DOC)

Table S1 **Baseline immobility time (FST_BAS_) of 5 inbred mouse strains.**
(DOC)

Table S2 **Locomotor activity of B6 and FVB treated with saline or 20 mg/kg of fluoxetine in the open field test.**
(DOC)

Table S3 **FST immobility time for B6, FVB and their F1, F2 generation mice after fluoxetine treatments (20 mg/kg) (FST_FLX_).**
(DOC)

Table S4 **mRNA expression levels of cyclophilin A (mean Ct ± SD) in the brain regions of C57BL/6J and FVB/NJ after treatment with fluoxetine (20 mg/kg) or saline.**
(DOC)

Table S5 **Clinical and demographic characteristics of the MDD patients and their responses to antidepressant treatments.**
(DOC)
