# Supplementary material for: Convergent Evidence from Mouse and Human Studies Suggests the Involvement of Zinc Finger Protein 326 Gene in Antidepressant Treatment Response
Source: PLoS One. 2012 May 30;7(5):e32984. doi: 10.1371/journal.pone.0032984 (PMC3364255; doi:10.1371/journal.pone.0032984)
Supplement: Figure S1 — LOD scores for linkage for z_ FSTFLX. The solid blue line at LOD = 3.38 and green dash line at LOD = 4.08 denotes the genome-wide significant threshold for z_ FSTFLX (SIM) and z_ FSTFLX (CIM) respectively. The gray zone indicates the 2-LOD confidence interval. z_ FSTFLX: the baseline adjusted FST immobility with fluoxetine treatment; LOD: logarithm of odds; SIM: simple interval mapping; CIM: composite interval mapping. (DOC) [file pone.0032984.s001.doc]

**Figure S1**: LOD scores for linkage for z_ FST_FLX_. The solid blue line at LOD = 3.38 and green dash line at LOD = 4.08 denotes the genome-wide significant threshold for z_ FST_FLX_ (SIM) and z_ FST_FLX_ (CIM) respectively. The gray zone indicates the 2-LOD confidence interval. z_ FST_FLX_: the baseline adjusted FST immobility with fluoxetine treatment; LOD: logarithm of odds; SIM: simple interval mapping; CIM: composite interval mapping.


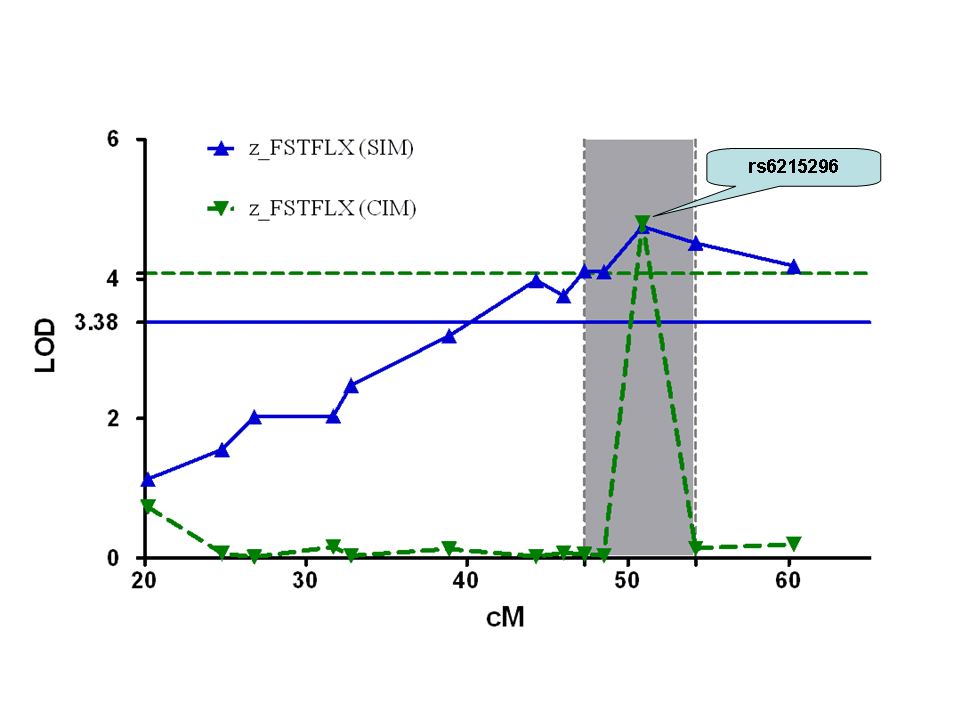


**Supplementary figure 2**: Cartoons indicate that different alleles ofrs33550587 (G>A) (5a) and rs13473815 (G>A) may have different effects on the secondary structure of transcribed *Zfp326* mRNA (5b).

5a).


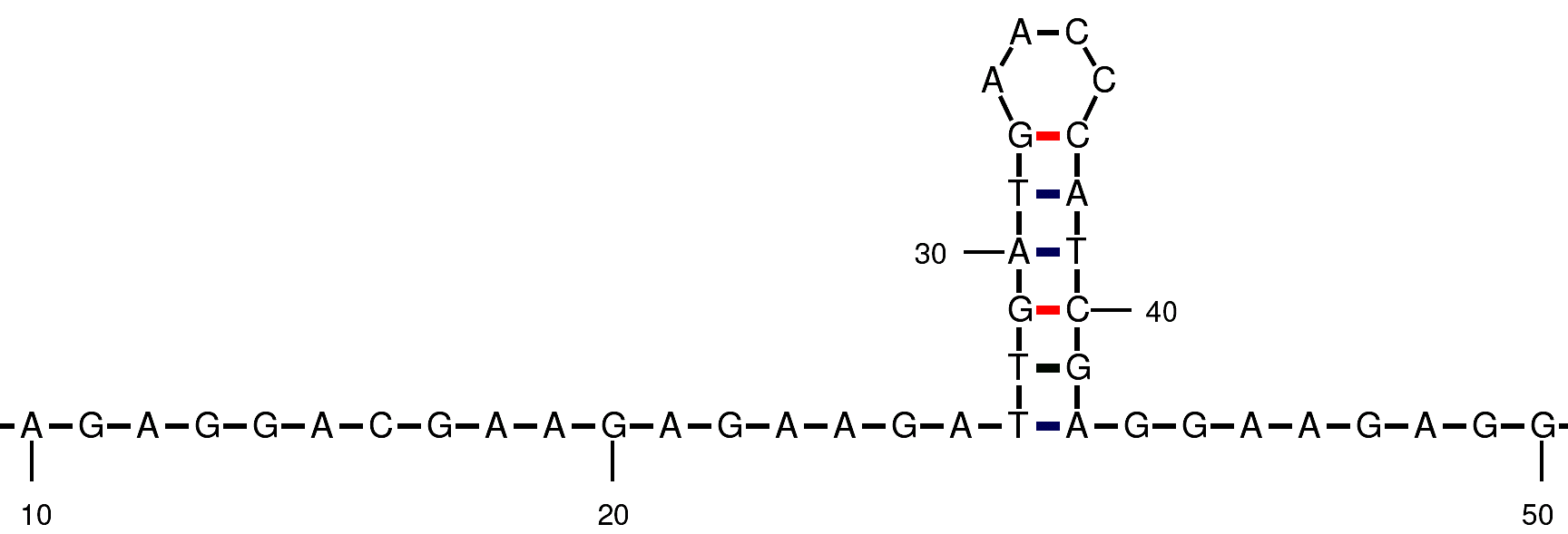


5b).
