# Supplementary material for: Convergent Evidence from Mouse and Human Studies Suggests the Involvement of Zinc Finger Protein 326 Gene in Antidepressant Treatment Response
Source: PLoS One. 2012 May 30;7(5):e32984. doi: 10.1371/journal.pone.0032984 (PMC3364255; doi:10.1371/journal.pone.0032984)
Supplement: Figure S2 — Cartoons indicate that different alleles ofrs33550587 (G>A) (5a) and rs13473815 (G>A) may have different effects on the secondary structure of transcribed Zfp326 mRNA (5b). (DOC) [file pone.0032984.s002.doc]

**Figure S2**: Cartoons indicate that different alleles ofrs33550587 (G>A) (5a) and rs13473815 (G>A) may have different effects on the secondary structure of transcribed *Zfp326* mRNA (5b).

5a).


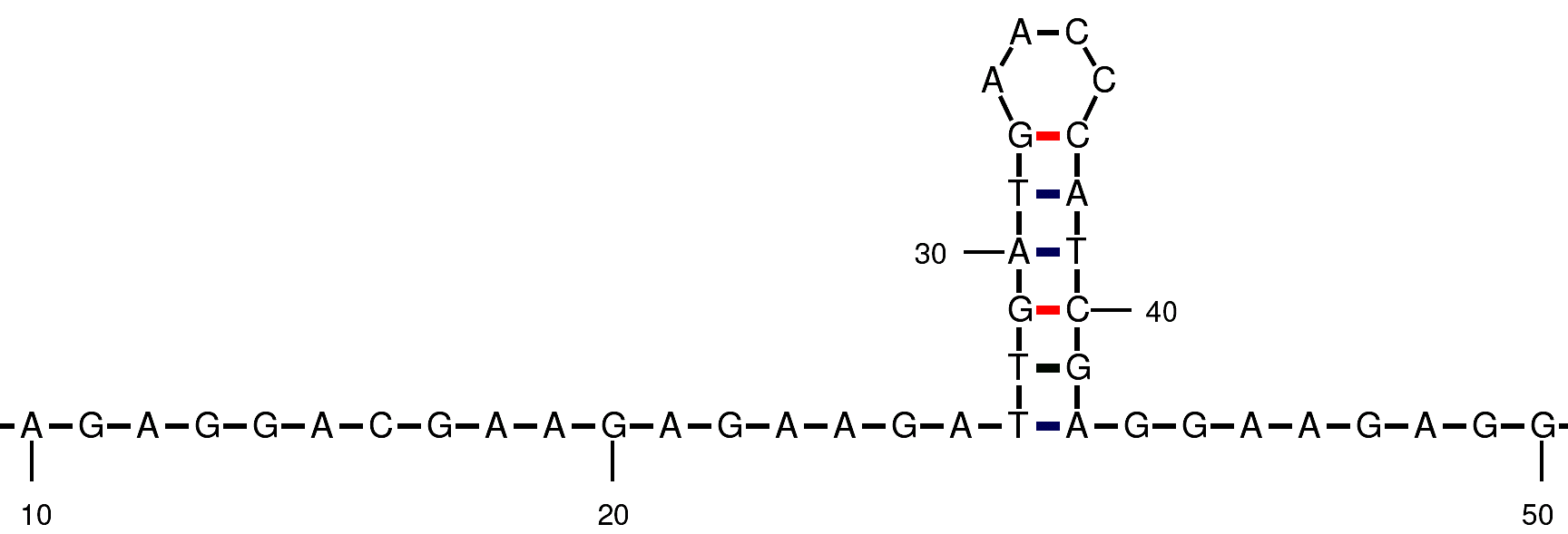


5b).
