# Supplementary material for: Convergent Evidence from Mouse and Human Studies Suggests the Involvement of Zinc Finger Protein 326 Gene in Antidepressant Treatment Response
Source: PLoS One. 2012 May 30;7(5):e32984. doi: 10.1371/journal.pone.0032984 (PMC3364255; doi:10.1371/journal.pone.0032984)
Supplement: Table S1 — Baseline immobility time (FSTBAS) of 5 inbred mouse strains. (DOC) [file pone.0032984.s003.doc]

**Table S1:** Baseline immobility time (FST_BAS_) of 5 inbred mouse strains

| Strain | N | Mean ± SD (sec) |
| --- | --- | --- |
| BALB/cByJ | 48 | 220.2 ± 9.5 |
| C57BL/6J | 42 | 196.6 ± 19.7 |
| FVB/NJ | 54 | 196.6 ± 15.1 |
| DBA/2N | 43 | 188.7 ± 33.3 |
| C3H/HeN | 45 | 177.3 ± 30.7 |
