# Supplementary material for: Convergent Evidence from Mouse and Human Studies Suggests the Involvement of Zinc Finger Protein 326 Gene in Antidepressant Treatment Response
Source: PLoS One. 2012 May 30;7(5):e32984. doi: 10.1371/journal.pone.0032984 (PMC3364255; doi:10.1371/journal.pone.0032984)
Supplement: Table S2 — Locomotor activity of B6 and FVB treated with saline or 20 mg/kg of fluoxetine in the open field test. (DOC) [file pone.0032984.s004.doc]

**Table S2**: Locomotor activity of B6 and FVB treated with saline or 20 mg/kg of fluoxetine in the open field test.

| Strain | Treatment | Distance, cm* | P value | Traveling time, sec* | P value | Speed, cm/sec* | P value |
| --- | --- | --- | --- | --- | --- | --- | --- |
| B6 | Control (n = 10) | 4631.7 ± 2108.8 | 0.912 | 181.6 ± 77.5 | 0.889 | 24.9 ± 2.2 | 0.910 |
|  | 20 mg/kg (n = 10) | 4530.2 ± 1935.3 |  | 176.9 ± 70.2 |  | 25.0 ± 2.2 |  |
| FVB | Control (n = 11) | 5063.6 ± 660.4 | 0.274 | 195.4 ± 29.8 | 0.572 | 26.1 ± 2.0 | 0.193 |
|  | 20 mg/kg (n = 12) | 4637.3 ± 1086.4 |  | 185.8 ± 47.0 |  | 25.1 ± 1.6 |  |

* Data are presented as mean ± standard deviation.
