# Supplementary material for: Convergent Evidence from Mouse and Human Studies Suggests the Involvement of Zinc Finger Protein 326 Gene in Antidepressant Treatment Response
Source: PLoS One. 2012 May 30;7(5):e32984. doi: 10.1371/journal.pone.0032984 (PMC3364255; doi:10.1371/journal.pone.0032984)
Supplement: Table S3 — FST immobility time for B6, FVB and their F1, F2 generation mice after fluoxetine treatments (20 mg/kg) (FSTFLX). (DOC) [file pone.0032984.s005.doc]

**Table S3**: FST immobility time for B6, FVB and their F1, F2 generation mice after fluoxetine treatments (20 mg/kg) (FST_FLX_).

|  | Mean ± SD (sec) | Observed variance | Variance (additive, V_A_)^*^ | Variance (dominant, V_D_)^*^ |
| --- | --- | --- | --- | --- |
| B6 (n = 19) | 214.7 ± 13.7 | 187.8 |  |  |
| F1 (n = 112) | 205.4 ± 24.5 | 602.4 |  |  |
| FVB (n = 22) | 165.1 ± 26.6 | 705.9 |  |  |
| F2 (n = 865) | 195.0 ± 32.2 | 1039.7 | 307.5 | 60.1 |

SD: standard deviation.

* The detailed description for the estimation of V_A_ and V_D_ can be seen in Falconer & Mackay (Falconer & Mackay 1996).
