# Supplementary material for: Convergent Evidence from Mouse and Human Studies Suggests the Involvement of Zinc Finger Protein 326 Gene in Antidepressant Treatment Response
Source: PLoS One. 2012 May 30;7(5):e32984. doi: 10.1371/journal.pone.0032984 (PMC3364255; doi:10.1371/journal.pone.0032984)
Supplement: Table S4 — mRNA expression levels of cyclophilin A (mean Ct ± SD) in the brain regions of C57BL/6J and FVB/NJ after treatment with fluoxetine (20 mg/kg) or saline. (DOC) [file pone.0032984.s006.doc]

**Table S4**: mRNA expression levels of cyclophilin A (mean Ct ± SD) in the brain regions of C57BL/6J and FVB/NJ after treatment with fluoxetine (20 mg/kg) or saline.

| Region | FC | | Hip | | HTh | | Amy | | NAc | | Th | | ST | |
| --- | --- | --- | --- | --- | --- | --- | --- | --- | --- | --- | --- | --- | --- | --- |
|  | FLX | SAL | FLX | SAL | FLX | SAL | FLX | SAL | FLX | SAL | FLX | SAL | FLX | SAL |
| C57BL/6J | 19.6±0.5 | 19.6±0.3 | 19.9±0.5 | 19.9±0.5 | 21.2±0.3 | 21.2±0.3 | 20.1±0.5 | 20.3±0.6 | 20.0±0.7 | 20.3±0.6 | 20.5±0.3 | 20.5±0.3 | 21.2±0.5 | 21.2±0.2 |
| FVB/NJ | 19.9±0.5 | 19.9±0.5 | 20.1±0.4 | 20.1±0.3 | 21.3±0.4 | 21.3±0.6 | 20.4±0.3 | 20.6±0.3 | 20.1±0.3 | 20.1±0.3 | 20.9±0.6 | 20.7±0.5 | 21.7±0.4 | 21.5±0.4 |

Ct: polymerase chain reaction cycle at which the sample reached the threshold; SD: standard deviation; FLX: fluoxetine. FC: frontal cortex; Hip: hippocampus; HTh: hypothalamus; Amy: amygdala; NAc: nucleus accumbens; Th: thalamus; St: striatum.
