# Supplementary material for: Convergent Evidence from Mouse and Human Studies Suggests the Involvement of Zinc Finger Protein 326 Gene in Antidepressant Treatment Response
Source: PLoS One. 2012 May 30;7(5):e32984. doi: 10.1371/journal.pone.0032984 (PMC3364255; doi:10.1371/journal.pone.0032984)
Supplement: Table S5 — Clinical and demographic characteristics of the MDD patients and their responses to antidepressant treatments. (DOC) [file pone.0032984.s007.doc]

**Table S5**: Clinical and demographic characteristics of the MDD patients and their responses to antidepressant treatments.

| Treatment duration |  | Age (SD), years | Gender, M/F | Antidepressant, Flu/Cip | Baseline HAMD (SD) | Current episode duration, months (SD) | Number of previous episodes (SD) |
| --- | --- | --- | --- | --- | --- | --- | --- |
| 8 weeks | Responder (n = 172) | 46.8 (14.9) | 71/101 | 84/88 | 27.8 (4.1) | 12.2 (13.9) | 1.2 (0.5) |
|  | Non-responder (n = 90) | 46.8 (14.8) | 34/56 | 52/38 | 27.6 (4.2) | 12.1 (13.2) | 1.4 (1.1) |
